# Depth for Underwater Acoustic Detection in Deep-Sea (>5000 m) Complex Marine Environments Based on the Bellhop Model

**DOI:** 10.3390/s26103149

**Published:** 2026-05-15

**Authors:** Xiaofang Sun, Shisong Zhang, Pingbo Wang

**Affiliations:** 1 Naval University of Engineering, Wuhan 430033, China; 2 Dalian Naval Academy, Dalian 116018, China

**Keywords:** underwater remote sensing, buoy deployment, transceiver depth difference, safety margin, Bellhop model, acoustic reciprocity

## Abstract

**Highlights:**

**What are the main findings?**

- Acoustic reciprocity is validated in deep-sea (>5000 m) complex seamount terrains with a maximum relative error of <1.2%, enabling simplified bidirectional transmission loss calculations for large-scale network planning, which expands its applicable boundary from simple waveguides to complex deep-sea environments; this enables the development of a novel, equivalent, superposition modeling framework for bidirectional transmission loss (TL), simplifying large-scale network planning calculations and filling the gap of quantitative verification in deep-sea complex environments.
- A critical non-linear sensitivity mechanism was identified for mid-layer sources (160–350 m), in which the detection range drops by ~50% and near-field coverage fragments when the transceiver depth difference exceeds a static threshold of ~185 m.
- The equivalent bidirectional TL modeling framework converts hard-to-calculate target-to-receiver TL into receiver-to-target TL, simplifying two-way TL calculation for transceivers at different positions to the superposition of TL from two sources at the same grid point.

**What are the implications of the main findings?**

- A robust engineering threshold of 150 m for transceiver depth difference is established by incorporating a safety margin against dynamic environmental fluctuations (e.g., internal waves), preventing catastrophic performance collapse in operational scenarios and filling the industry gap in quantitative deployment design for deep-sea acoustic detection.
- A layered deployment protocol is proposed, defining a 160–350 m “Optimal Detection Window” for long-range tasks while mandating strict depth constraints (<150 m) to ensure continuous near-field coverage and reliable 3D observation network design; the protocol provides flexible depth tolerance for shallow/deep sources, supporting reliable 3D observation network design.

**Abstract:**

Quantifying the detection efficiency of buoy-based sonar and optimizing deployment strategies in complex marine environments remain significant challenges. This study proposes a transceiver depth optimization method based on the Bellhop ray model to enhance underwater remote sensing data quality. For the first time, we validated the applicability of acoustic reciprocity in deep-sea environments exceeding 5000 m, characterized by non-uniform sound speed profiles, horizontal inhomogeneity, and steep seamount terrain, with a maximum relative error of <1.2%. This extends the applicable boundaries of the acoustic reciprocity theorem from idealized simple waveguides to complex, realistic deep-sea environments. Building on this validation, we developed a novel, equivalent, superposition modeling framework for bidirectional transmission loss (TL), which converts the computationally intractable TL from target to receiver into the calculable TL from receiver to target, thus significantly reducing computational complexity. Systematic simulations uncovered a depth-layered dependency mechanism: shallow sources (23.14~69.42 m) and deep sources (≥347.10 m) show robustness to large depth differences exceeding 500 m, whereas mid-layer sources (161.98~231.40 m) exhibit a distinct critical threshold effect. Static simulations identify a performance degradation cliff with an onset at an approximate depth difference of 185 m, leading to a 50% reduction in detection range and fragmented near-field detection coverage. To accommodate environmental temporal variability (e.g., internal waves), a conservative safety margin was incorporated, establishing a robust engineering threshold of 150 m. Accordingly, we define 160~350 m as the optimal detection depth window and propose a layered deployment protocol that fills a critical industry gap in quantitative deployment design for deep-sea acoustic detection. Specifically, transceiver depth differences should be strictly constrained to <150 m for mid-layer operations, while more-flexible depth configurations are permissible for shallow and deep sources. These findings furnish quantitative engineering criteria for the design of reliable underwater remote sensing networks, while balancing long-range detection stability and near-field coverage integrity.

## 1. Introduction

Buoy systems are the core equipment for underwater acoustic detection in deep-sea (>5000 m) complex marine environments, which play an irreplaceable role in long-range target monitoring, three-dimensional marine observation and underwater security operations [1]. The transceiver depth configuration is the key factor affecting the detection performance of buoy sonar, but there is still a lack of quantitative mechanism, critical threshold and standardized deployment protocol for depth optimization in engineering practice. The detection range of buoy sonar is determined by the sonar equation, and Transmission Loss (TL) is the core parameter of acoustic energy attenuation [2]. For fixed system parameters, the effective detection condition can be simplified as the sum of two-way TL lower than a specific threshold, so the spatial distribution of effective detection points is the core basis for optimizing deployment schemes.

The acoustic reciprocity theorem can effectively simplify the TL calculation of large-scale detection networks, but its quantitative applicability in deep-sea environments with coupled horizontal inhomogeneous sound speed and steep seamount topography has not been verified [3]. Existing studies only confirm the reciprocity in simple waveguides, ignoring the complex hydrological and topographic coupling effects in actual deep-sea engineering, which leads to the unreliability of reciprocity-based TL calculation in complex scenarios [4,5].

At present, the research on buoy deployment mainly focuses on the detection range analysis of specific working conditions [6,7,8,9,10], and there is a lack of systematic comparison of detection spatial distribution between co-depth and bistatic depth configurations. More importantly, the non-linear sensitivity mechanism of transceiver depth difference, the critical threshold of performance collapse and the engineering-oriented deployment strategy have not been revealed and proposed, resulting in the absence of quantitative criteria for deep-sea buoy depth deployment [11,12].

To fill the above research gaps, this paper carries out research on transceiver depth optimization for deep-sea acoustic detection. The core innovations of this paper are as follows: ① Quantitatively verify the acoustic reciprocity in deep-sea complex seamount terrain with a maximum relative error less than 1.2%, which provides a reliable theoretical basis for TL simplification calculation. ② Reveal the depth-layered dependency mechanism of detection performance and find the static critical threshold (185 m) and engineering safety threshold (150 m) of mid-layer source depth difference. ③ Construct the optimal detection depth window (160–350 m) and propose a layered deployment protocol for deep-sea buoys. In this study, the TL calculation and simulation analysis are carried out based on Bellhop model, HYCOM hydrological data and ETOPO1 topographic data, with *TL_sum* ≤ 160 dB as the effective detection criterion. This research is only applicable to deep-sea complex environments with water depth >5000 m, horizontal inhomogeneous sound speed profile and undulating seabed terrain, and it provides quantitative engineering guidelines for high-performance deployment of deep-sea underwater acoustic detection networks.

## 2. Methodology

### 2.1. Theoretical Framework

#### 2.1.1. Acoustic Reciprocity Theorem

In a linear, passive, and isotropic acoustic medium, the complex pressure P(A→B) measured at point *B* from a source at *A* equals P(B→A) when positions are swapped. Thus, ${TL}_{A\to B}={TL}_{B\to A}$. This principle allows the substitution of the difficult-to-calculate Target-to-Receiver loss with the Receiver-to-Target loss, streamlining the identification of effective detection points in large-scale simulations.

#### 2.1.2. Sonar Equation and Detection Criterion

The active sonar equation is expressed as

(1) $$FOM=SL-{TL}_{3}-TS-{TL}_{2}+NL-DI$$

where *SL*: source level; *TS*: target strength; *NL*: noise level (set to 60–70 dB according to Urick’s deep-sea ambient noise standard); *DI*: directivity index; ${TL}_{3}$/${TL}_{2}$: transmission loss from source to receiver/receiver to target.

Based on the verified reciprocity of acoustic propagation, a modeling process for equivalent superposition of two-way transmission loss is established: the transmission loss ${TL}_{1}$ from the target to the receiver is equivalently converted into the transmission loss ${TL}_{2}$ from the receiver to the target. The two-way transmission loss calculation for the bistatic sonar configuration is thus simplified to the superposition of transmission losses generated by two sound sources within the same computational grid.

TL is calculated by the following: HYCOM sound speed profile → Bellhop ray tracing simulation → acoustic energy attenuation statistics → TL value. Effective detection occurs when *FOM* ≥ 0 dB (signal is detectable) [3]. With calibrated equipment parameters (SL,TS,NL,DI), the criterion simplifies to ${TL}_{2}+{TL}_{3}\leq 160\;dB$. Spatial points satisfying this inequality constitute the effective detection volume, forming the basis for deployment design.

### 2.2. Verification of Acoustic Reciprocity in Complex Environments

#### 2.2.1. Environmental Model Construction

**Figure 1:**
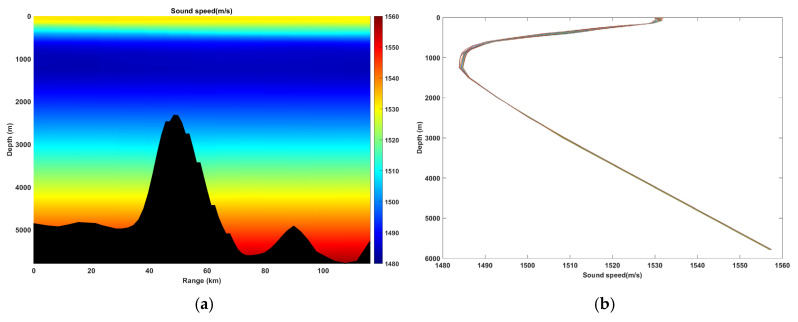
Sound speed profile and terrain distribution map. (**a**) Sound speed profile; (**b**) terrain distribution.

The study area is located in the waters east of 122.47°~123.60° E and south of 22.33° N, with a total span of 116.21 km water depth >5000 m (deep-sea environment). The hydrological data were sourced from the HYCOM global ocean model, and the bathymetric data were obtained from the ETOPO1 global relief model. The sound speed profile exhibits significant horizontal inhomogeneity, and the seabed topography is complex, featuring undulating seamounts and trenches. Based on these data, a two-dimensional acoustic propagation environment model was constructed (as shown in Figure 1).

#### 2.2.2. Verification of the Acoustic Reciprocity Theorem

The classical acoustic reciprocity theorem presupposes symmetric medium parameters and smooth boundaries. However, the study area is characterized by steep seamount topography (with significant slope variations) and horizontally inhomogeneous sound speed structures. In such environments, ray-based models are prone to generating singularities in caustic zones, and the angle-dependent nature of seabed reflection coefficients may induce subtle discrepancies in path sampling between the Source-to-Receiver (*A* → *B*) and Receiver-to-Source (*B* → *A*) directions. Theoretically, this poses a risk of reciprocity failure. This issue directly impacts the reliability of model simplification and computational efficiency in underwater acoustic detection data processing.

**Figure 2:**
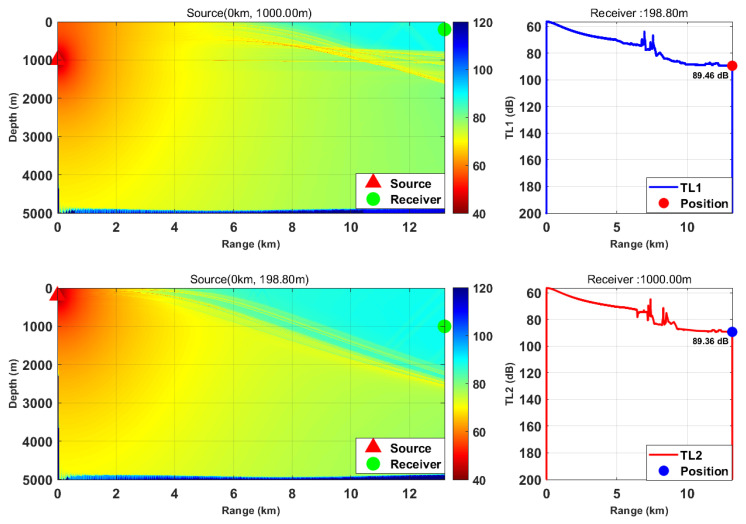
Comparison of transmission loss distribution from sound source to receiver at different receiving depths (distance: 13.19 km).

**Figure 3:**
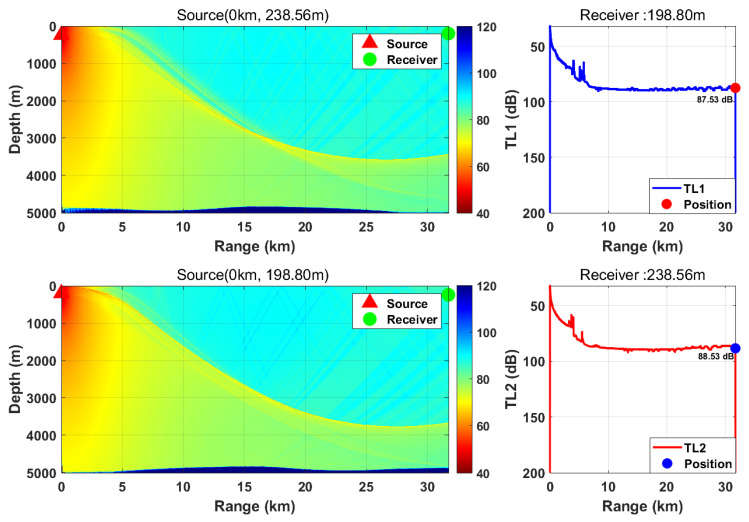
Comparison of transmission loss distribution from sound source to receiver at different receiving depths (distance: 31.73 km).

**Figure 4:**
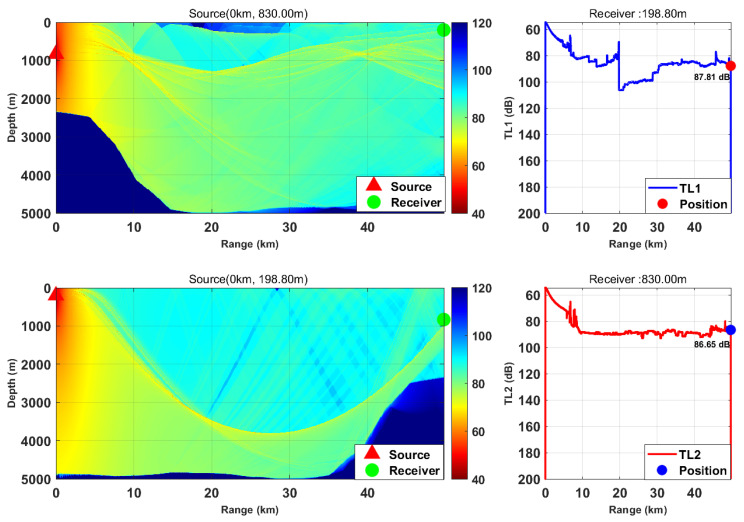
Comparison of transmission loss distribution from sound source to receiver at different receiving depths (distance: 49.76 km).

**Figure 5:**
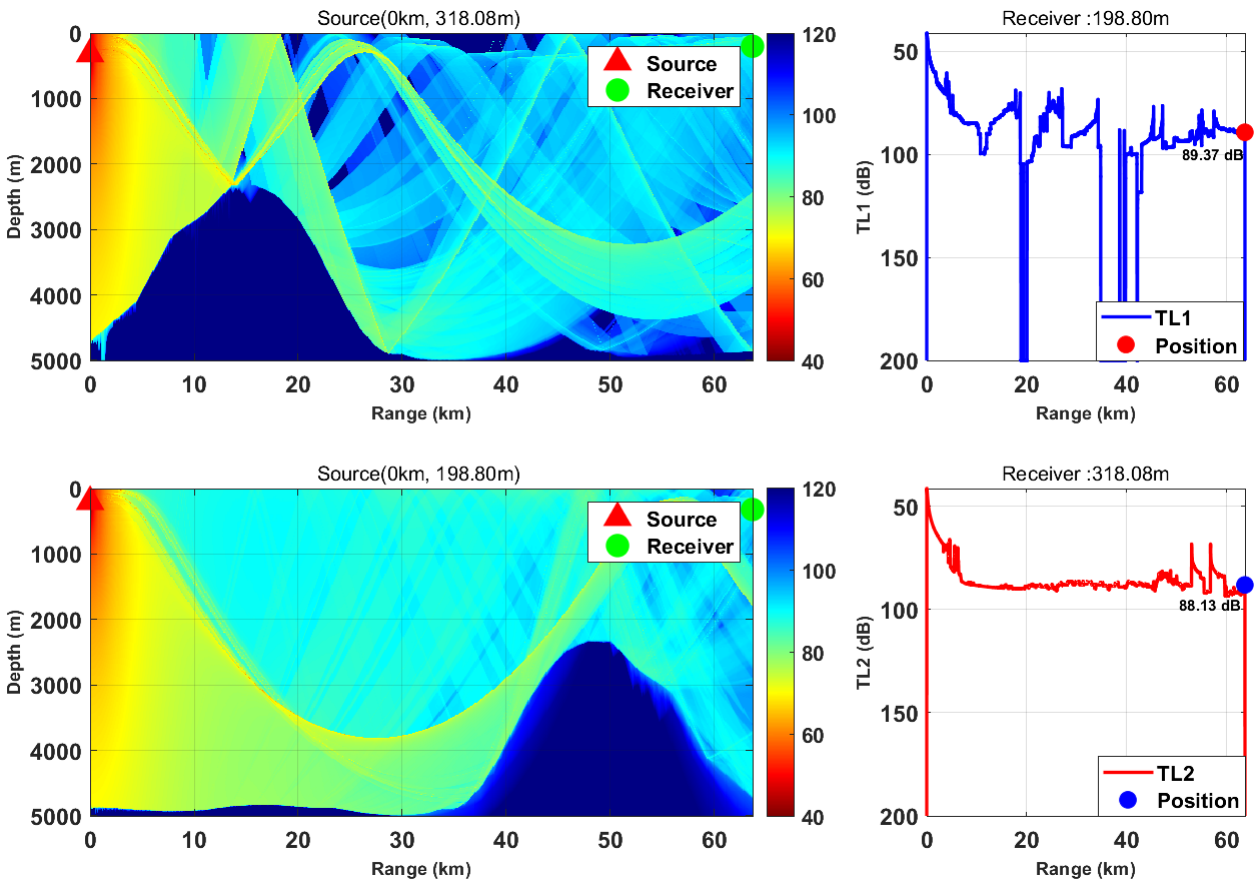
Comparison of transmission loss distribution from sound source to receiver at different receiving depths (distance: 63.67 km).

**Figure 6:**
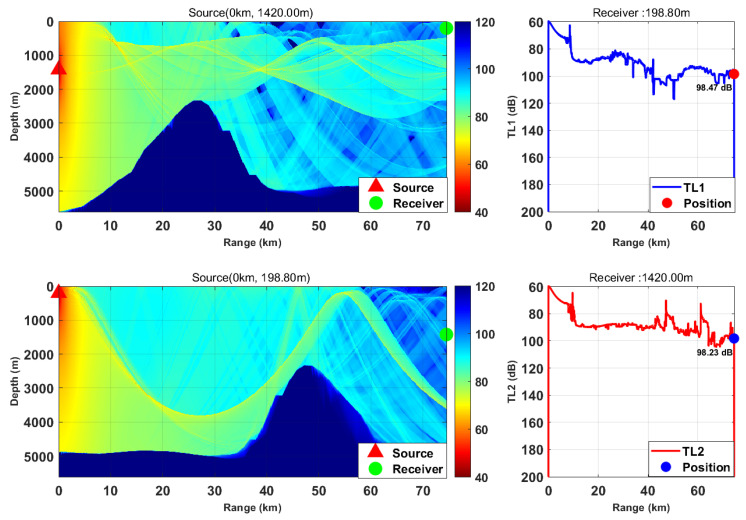
Comparison of transmission loss distribution from sound source to receiver at different receiving depths (distance: 74.51 km).

**Figure 7:**
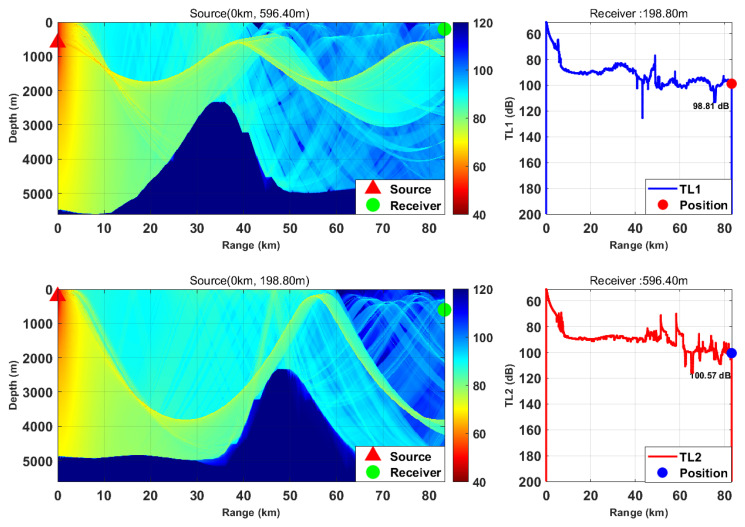
Comparison of transmission loss distribution from sound source to receiver at different receiving depths (distance: 83.24 km).

**Figure 8:**
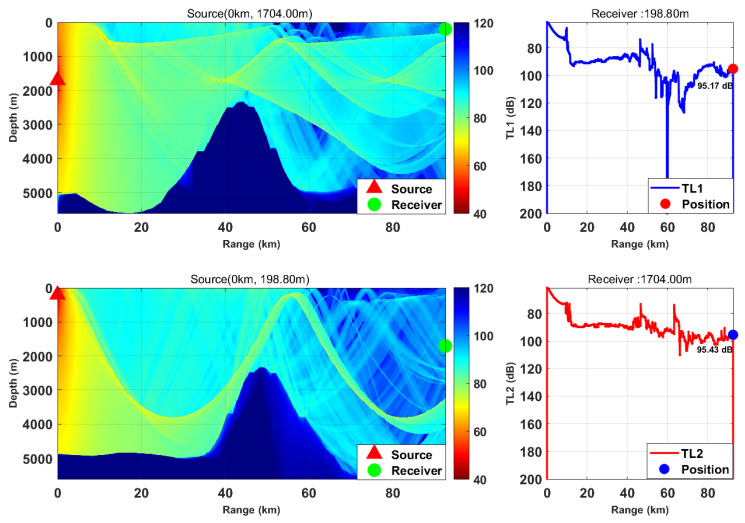
Comparison of transmission loss distribution from sound source to receiver at different receiving depths (distance: 92.51 km).

**Figure 9:**
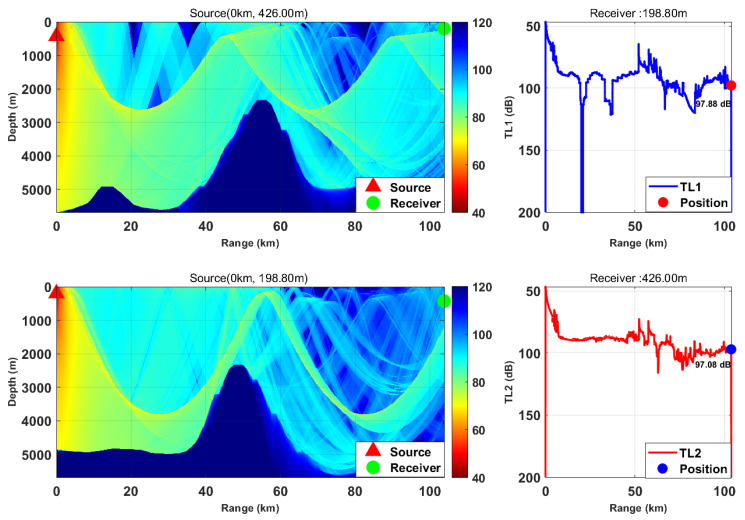
Comparison of transmission loss distribution from sound source to receiver at different receiving depths (distance: 103.85 km).

**Figure 10:**
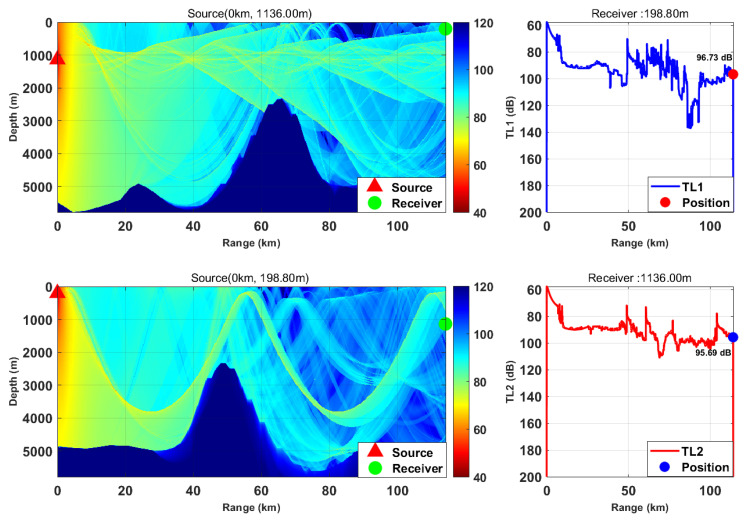
Comparison of transmission loss distribution from sound source to receiver at different receiving depths (distance: 114.15 km).

This study is based on the Bellhop model with a source frequency of 300 Hz; the 300 Hz frequency is selected because, for the deep-sea environment with water depth of more than 5000 m, it fully meets the high-frequency applicable conditions of Bellhop ray theory and belongs to the optimal low-frequency band for deep-sea long-range detection with slow acoustic attenuation and long propagation distance. We selected nine typical horizontal ranges and multiple transceiver depth combinations. We calculated the transmission loss from the source (target) to the receiver (${TL}_{1}$) and from the receiver to the source (target) (${TL}_{2}$) separately. By comparing the difference between these two values, we verified the applicability of the acoustic reciprocity theorem in complex marine environments. The results are presented in Figure 2, Figure 3, Figure 4, Figure 5, Figure 6, Figure 7, Figure 8, Figure 9 and Figure 10.

**Figure 11:**
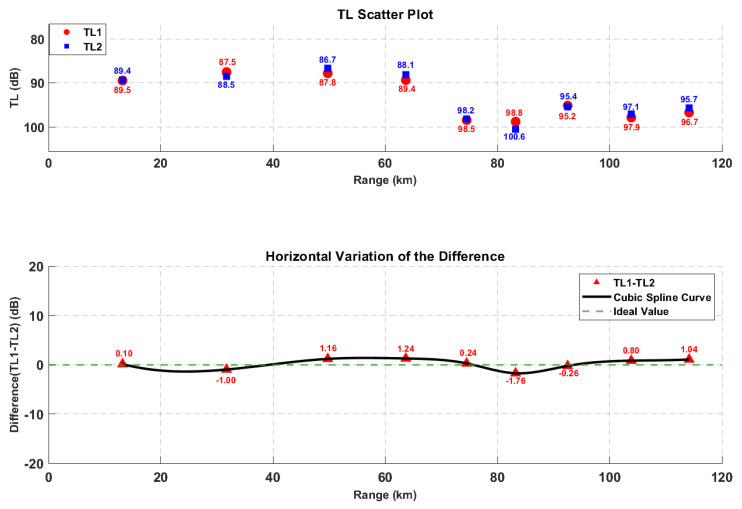
Variation trend of TL difference in the *x*-axis direction.

By constructing a bidirectional transmission loss comparison model, we defined the validity boundaries of the theorem under complex boundary conditions. The results indicate that, although terrain scattering and ray caustics introduce local phase perturbations, the macroscopic transmission loss remains highly symmetric. The maximum absolute deviation was found to be only 1.76 dB, with a relative error of less than 1.2% (see Figure 11). This finding confirms that, at the scale of engineering applications, complex topography does not destroy the macroscopic reciprocity characteristics of the sound field. This provides a solid physical basis for utilizing the reciprocity principle to simplify simulation calculations across multi-dimensional deployment spaces in subsequent steps. Furthermore, it holds significant importance for improving the efficiency of underwater acoustic detection data processing and constructing high-efficiency underwater acoustic detection networks.

### 2.3. Simulation Calculation of Effective Buoy Detection Range

Based on the acoustic field reciprocity theorem, the Bellhop model was employed to simulate and calculate transmission loss values for 21 distinct depth configurations. These configurations include

1. Co-depth deployments: Both source and receiver at identical depths (23.14 m, 69.42 m, 161.98 m, 231.40 m, 347.10 m, and 578.50 m).
2. Bistatic depth configurations: The source (target) was fixed at specific depths (23.14 m, 69.42 m, 161.98 m, 231.40 m, or 347.10 m), while the receiver was placed at varying depths.

Effective detection spatial points were screened using the criterion ${TL}_{2}+{TL}_{3}\leq 160\;dB$. Key performance indicators were extracted for each configuration, including the maximum detection range, the total number of effective points, and the number of effective points within the 0~300 m layer. These metrics were analyzed to elucidate the impact laws of depth combinations on underwater acoustic detection performance.

**Figure 12:**
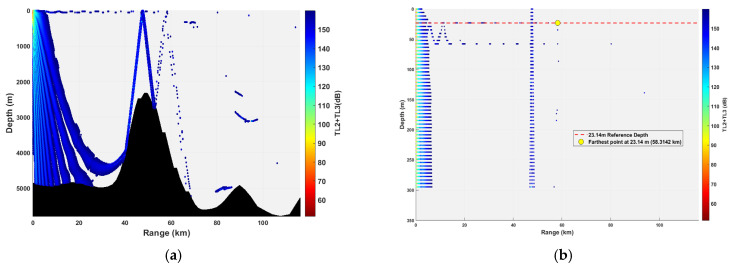
Spatial point distribution at transceiver depth of 23.14 m. (**a**) Distribution of all detectable spatial points; (**b**) distribution of detectable spatial points within 300 m.

**Figure 13:**
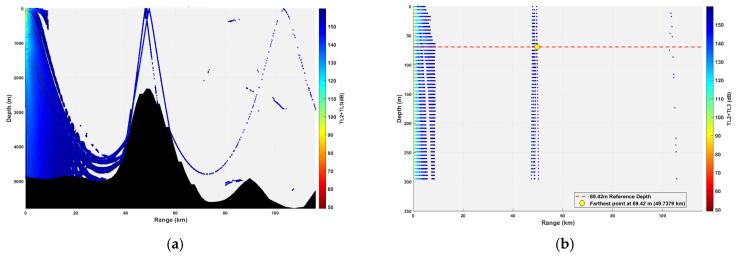
Spatial point distribution at transceiver depth of 69.42 m. (**a**) Distribution of all detectable spatial points; (**b**) distribution of detectable spatial points within 300 m.

**Figure 14:**
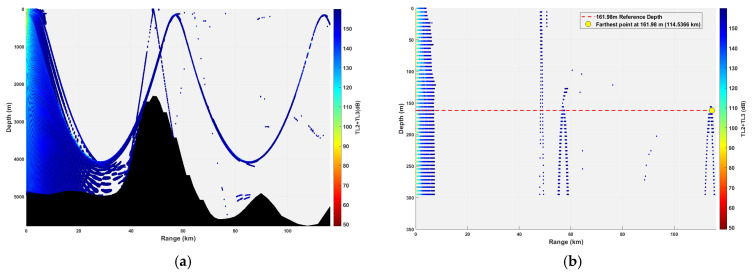
Spatial point distribution at transceiver depth of 161.98 m. (**a**) Distribution of all detectable spatial points; (**b**) distribution of detectable spatial points within 300 m.

**Figure 15:**
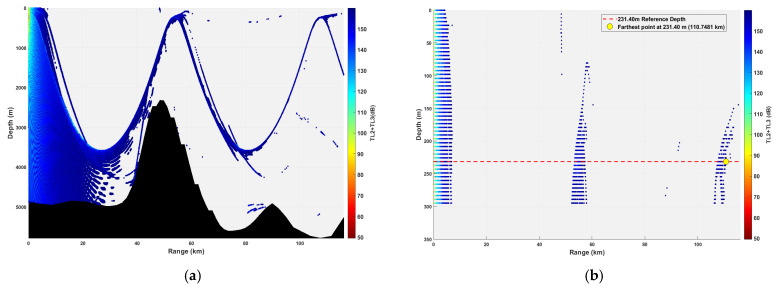
Spatial point distribution at transceiver depth of 231.40 m. (**a**) Distribution of all detectable spatial points; (**b**) distribution of detectable spatial points within 300 m.

**Figure 16:**
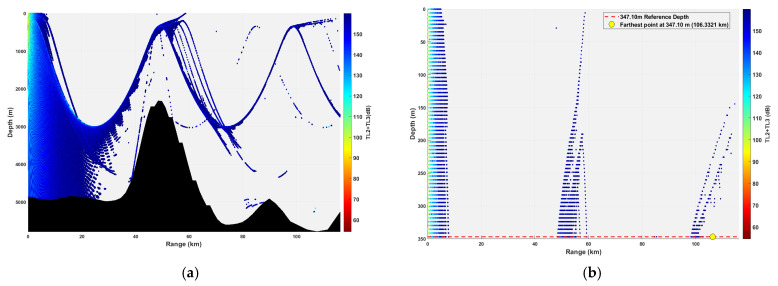
Spatial point distribution at transceiver depth of 347.10 m. (**a**) Distribution of all detectable spatial points; (**b**) distribution of detectable spatial points within 300 m.

**Figure 17:**
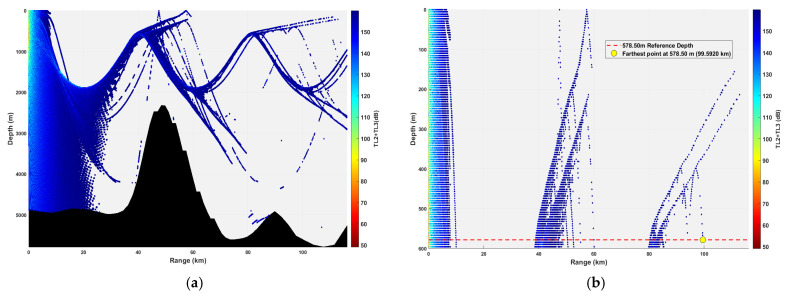
Spatial point distribution at transceiver depth of 578.50 m. (**a**) Distribution of all detectable spatial points; (**b**) distribution of detectable spatial points within 300 m.

**Figure 18:**
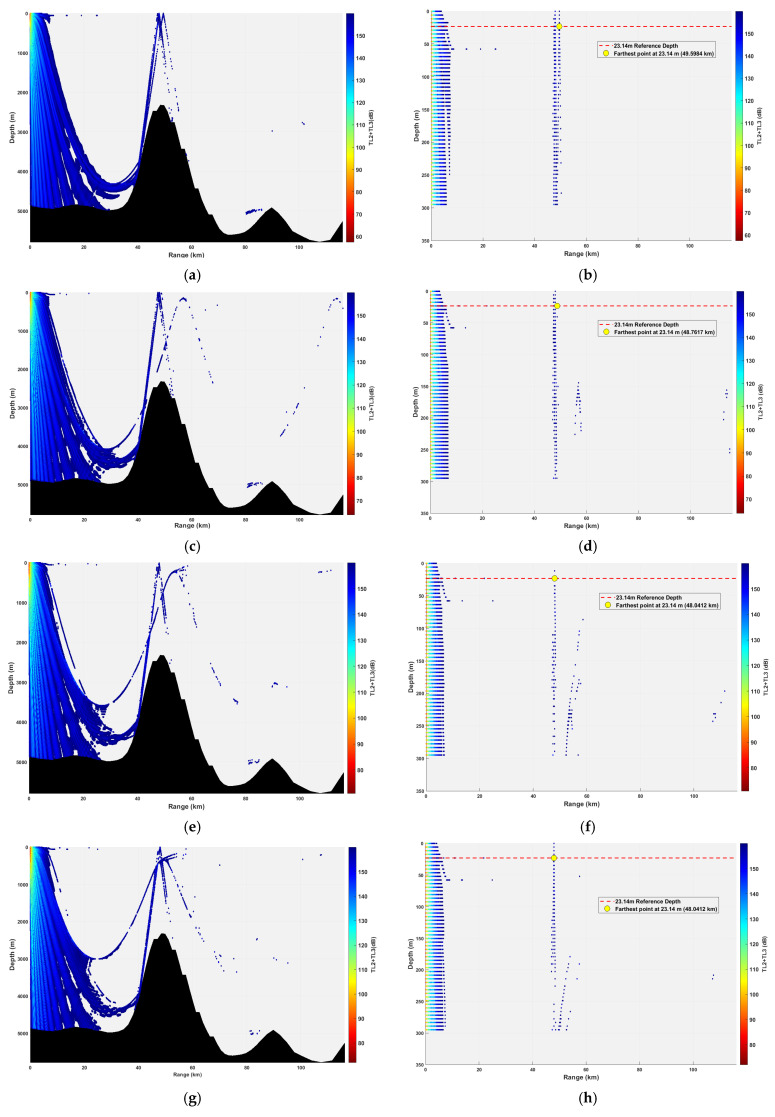

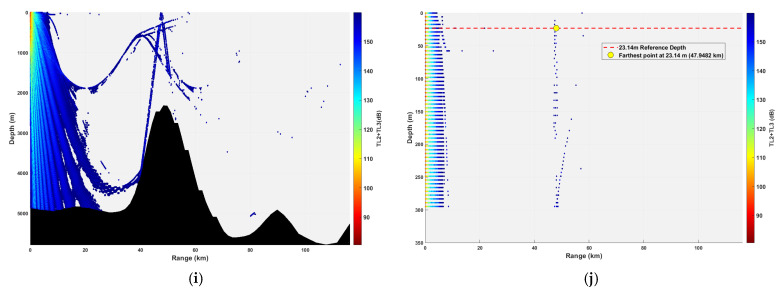
Spatial point distribution at a source depth of 23.14 m under different receiver depths. (**a**) Distribution of all spatial points at a receiver depth of 69.42 m; (**b**) spatial point distribution within 300 m at a receiver depth of 69.42 m; (**c**) Distribution of all spatial points at a receiver depth of 161.98 m; (**d**) spatial point distribution within 300 m at a receiver depth of 161.98 m; (**e**) Distribution of all spatial points at a receiver depth of 231.40 m; (**f**) spatial point distribution within 300 m at a receiver depth of 231.40 m; (**g**) Distribution of all spatial points at a receiver depth of 347.10 m; (**h**) spatial point distribution within 300 m at a receiver depth of 347.10 m; (**i**) Distribution of all spatial points at a receiver depth of 578.50 m; (**j**) spatial point distribution within 300 m at a receiver depth of 578.50 m.

**Figure 19:**
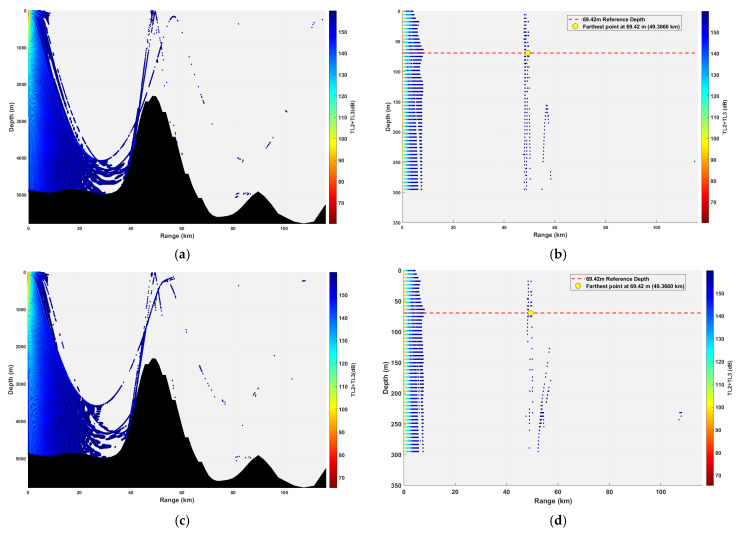

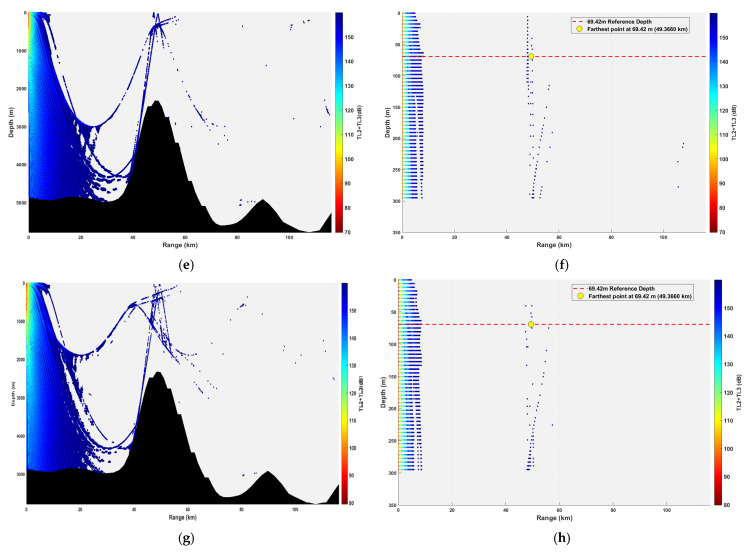
Spatial point distribution at sound source depth of 69.42 m and different receiver depths. (**a**) Distribution of all spatial points at receiver depth of 161.98 m; (**b**) distribution of spatial points at receiver depth of 161.98 m within 300 m; (**c**) distribution of all spatial points at receiver depth of 231.40 m; (**d**) distribution of spatial points at receiver depth of 231.40 m within 300 m; (**e**) distribution of all spatial points at receiver depth of 347.10 m; (**f**) distribution of spatial points at receiver depth of 347.10 m within 300 m; (**g**) distribution of all spatial points at receiver depth of 578.50 m; (**h**) distribution of spatial points at receiver depth of 578.50 m within 300 m.

**Figure 20:**
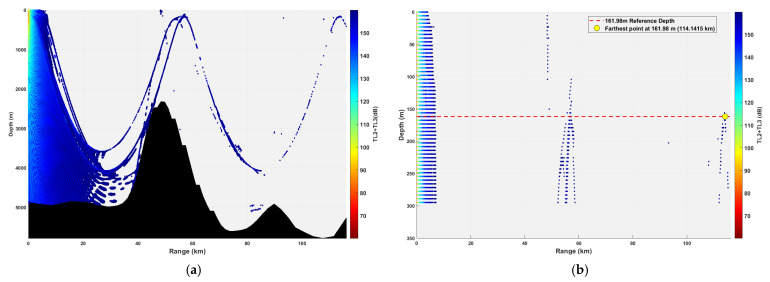

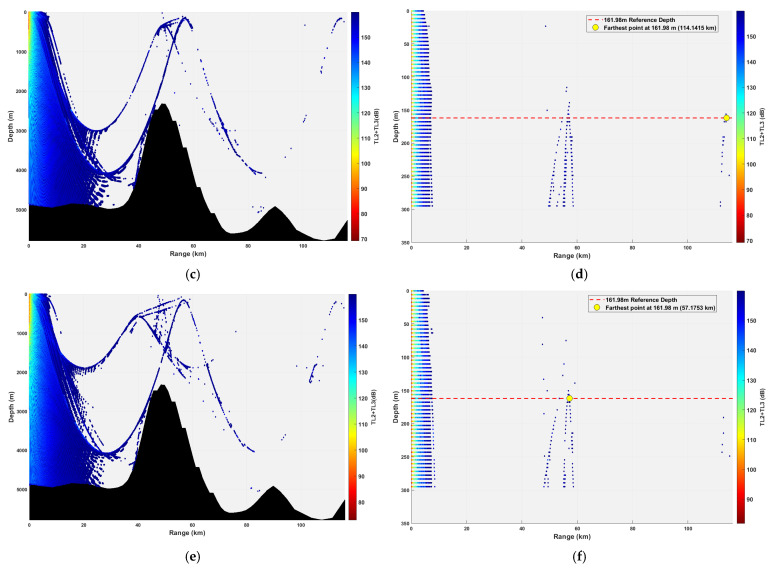
Spatial point distribution at sound source depth of 161.98 m and different receiver depths. (**a**) Distribution of all spatial points at receiver depth of 231.40 m; (**b**) distribution of spatial points at receiver depth of 231.40 m within 300 m; (**c**) distribution of all spatial points at receiver depth of 347.10 m; (**d**) distribution of spatial points at receiver depth of 347.10 m within 300 m; (**e**) distribution of all spatial points at receiver depth of 578.50 m; (**f**) distribution of spatial points at receiver depth of 578.50 m within 300 m.

**Figure 21:**
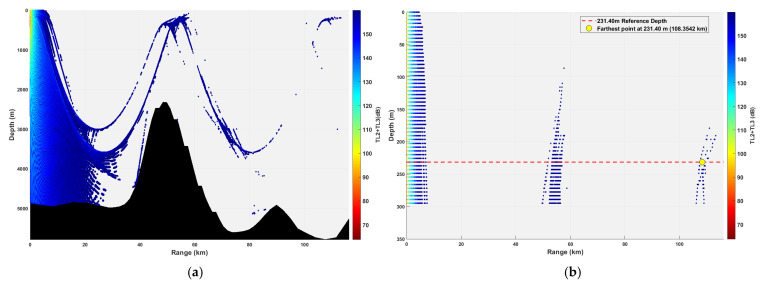

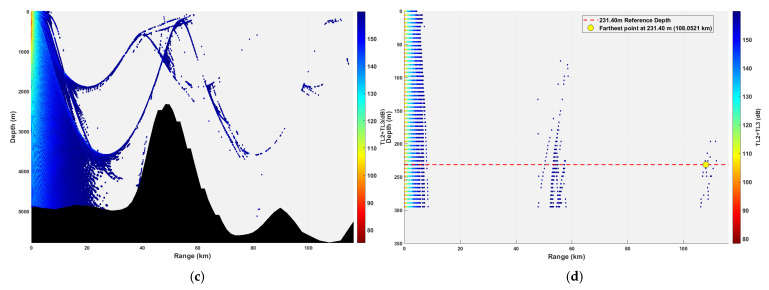
Spatial point distribution at sound source depth of 231.40 m and different receiver depths. (**a**) Distribution of all spatial points at receiver depth of 347.10 m; (**b**) distribution of spatial points at receiver depth of 347.10 m within 300 m; (**c**) distribution of all spatial points at receiver depth of 578.50 m; (**d**) distribution of spatial points at receiver depth of 578.50 m within 300 m.

**Figure 22:**
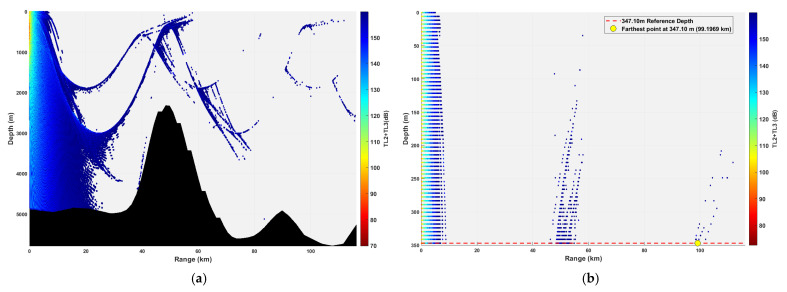
Spatial point distribution at sound source depth of 347.10 m and receiver depth of 578.50 m. (**a**) Distribution of all spatial points at receiver depth of 578.50 m; (**b**) distribution of spatial points at receiver depth of 578.50 m within 300 m.

In this study, the water column from 0 to 300 m is defined as the “Critical Detection Zone”. This definition is grounded in operational reality: Modern conventional submarines, when executing missions such as ambush, patrol, and weapon launch, operate predominantly within this depth interval. They utilize the thermocline for acoustic stealth and to maintain maneuvering advantages. Consequently, this layer represents the critical information acquisition region for underwater acoustic detection in specific application scenarios. The simulation results are illustrated in Figure 12, Figure 13, Figure 14, Figure 15, Figure 16, Figure 17, Figure 18, Figure 19, Figure 20, Figure 21 and Figure 22.

The marine detection area was discretized using a grid partitioning method to generate a spatial grid compatible with the Bellhop model output. A masking algorithm was then applied to screen and identify effective points satisfying the criterion ${TL}_{2}+{TL}_{3}\leq 160\;dB$. Statistical analysis methods were employed to compare the spatial distribution patterns, quantitative variations, and attenuation laws of the maximum detection range across different depth combinations. This analysis aims to elucidate the intrinsic correlation between buoy underwater acoustic detection performance and transceiver depth configurations. Furthermore, by integrating mechanisms of underwater acoustic propagation, we interpreted the physical essence underlying the impact of depth on detection performance. These findings provide robust theoretical support for optimizing buoy deployment schemes. The specific data are presented in Table 1.

## 3. Results and Analysis

### 3.1. Analysis of Acoustic Reciprocity Theorem Verification

The difference between ${TL}_{1}$ and ${TL}_{2}$ under different horizontal distances and transceiver depth combinations is shown in Figure 11. The results indicate that, in the complex marine environment, the minimum difference between ${TL}_{1}$ and ${TL}_{2}$ is 0.1 dB, observed over approximately flat terrain at a receiver depth of 1000 m. The maximum difference was 1.76 dB (relative error < 1.2%), occurring in the shadow zone behind seamount topography at a receiver depth of 596.4 m. The horizontal range of 40~65 km corresponds to the seamount influence zone, in which the transmission loss difference was relatively large (approximately 1.2 dB). However, compared with the magnitude of the transmission loss itself (80~100 dB), the difference between ${TL}_{1}$ and ${TL}_{2}$ is negligible. This demonstrates that the acoustic reciprocity theorem maintains engineering applicability in complex environments characterized by horizontally inhomogeneous sound speed profiles and undulating seabed topography. This finding provides a solid physical basis for leveraging the reciprocity theorem to significantly simplify simulation calculations across multi-dimensional deployment spaces, holding great significance for improving the efficiency of underwater acoustic detection data processing and constructing high-efficiency underwater acoustic detection networks.

### 3.2. Buoy Remote Sensing Performance Under Co-Depth Deployment

The spatial distribution of effective detection points for six co-depth configurations is shown in Figure 12, Figure 13, Figure 14, Figure 15, Figure 16 and Figure 17, with key performance metrics summarized in Table 1. The analysis yields the following insights:

1. Shallow Sources (23.14~69.42 m): The sound speed profile exhibits a positive gradient, causing acoustic rays to refract sharply towards the sea surface. This leads to rapid acoustic energy attenuation. Consequently, the maximum remote sensing detection range is limited to approximately 50 km, and the number of effective points within the 0~300 m layer is low (14,659 points at 23.14 m; 16,801 points at 69.42 m). Long-range underwater acoustic detection capabilities are thus constrained.
2. Mid-Layer Sources (161.98~231.40 m): This represents the optimal detection depth interval for buoy remote sensing. The sound speed profile shows a negative gradient with gentle variations, resulting in stable ray propagation paths with minimal energy loss from sea surface or seabed boundaries. Leveraging the ducting effect (waveguide mechanism), these configurations achieve long-range propagation, with maximum detection ranges exceeding 100 km (114.54 km at 161.98 m; 110.75 km at 231.40 m). The total number of effective spatial points exceeds 500,000, and the distribution within the 0~300 m layer is continuous, demonstrating superior short-range coverage performance.
3. Deep Sources (≥347.10 m): As depth increases, the influence of seamount topography intensifies, leading to increased reflection losses. The maximum detection range decreases slightly (106.33 km at 347.10 m; 99.59 km at 578.50 m). However, the total number of effective points remains high (616,947 points at 578.50 m), indicating that overall remote sensing performance remains stable.

Furthermore, the relationship between buoy remote sensing performance and deployment depth within the upper 500 m is non-linear rather than monotonically increasing or decreasing. Arbitrarily increasing deployment depth does not enhance performance and may even lead to range degradation due to increased reflection losses. This underscores the critical importance of optimizing deployment depth for underwater acoustic detection platforms.

### 3.3. Buoy Remote Sensing Performance Under Bistatic Depth Configurations

The spatial distribution of effective detection points for five fixed source depths combined with multiple receiver depths is presented in Figure 18, Figure 19, Figure 20, Figure 21 and Figure 22, with key metrics in Table 1. The analysis reveals the following:

1. Negative Correlation: There is a strong negative correlation between transceiver depth separation (Δz) and remote sensing performance. As Δz increases, the number of effective points decreases, and the distribution within the 0~300 m layer transitions from continuous to discrete patchy patterns. The effectively covered water column becomes thinner, directly compromising the integrity and continuity of underwater acoustic detection data.
2. Low Sensitivity of Shallow Sources: Shallow sources exhibit low sensitivity to depth separation. As Δz increases from 46.28 m to 555.36 m, the maximum detection range remains stable at 47~50 km, with no significant performance degradation.
3. High Sensitivity of Mid-Layer Sources: Mid-layer sources are highly sensitive to depth separation. When Δz exceeds 400 m, the maximum detection range plummets by approximately 50% (dropping from 114.54 km to 57.18 km for the 161.98 m source), resulting in a complete loss of long-range remote sensing capability.
4. Reduced Sensitivity of Deep Sources: Deep sources show reduced sensitivity. When paired with deeper receivers (e.g., 578.50 m), the maximum detection range remains above 99 km, demonstrating stability comparable with that in mid-layer co-depth scenarios.

Further analysis indicates that the core mechanism for this performance degradation is the spatial separation of the Source-to-Target and Receiver-to-Target propagation paths as Δz increases. This separation narrows the overlap of low-transmission-loss zones, thereby thinning the jointly effective water column.

### 3.4. Threshold Effect of Transceiver Depth Separation

Statistical analysis of detection performance metrics across all transceiver depth combinations reveals the following: While static simulation data indicates that significant performance degradation begins only when the depth difference reaches approximately 185 m, this study incorporates a conservative safety margin to account for environmental time-variance (e.g., internal waves and thermocline fluctuations) that can exacerbate acoustic field instability in real-world scenarios. Consequently, the engineering critical threshold is established at 150 m. The validation results demonstrate that

1. When the depth difference is ≤150 m: The superimposed distribution of ${TL}_{2}$ and ${TL}_{3}$ shows no significant variation. The attenuation rates for both the number of effective points and the maximum detection range remain below 5%, indicating that the buoy underwater remote sensing performance remains fundamentally stable and robust.
2. When the depth difference exceeds 150 m: The overlapping region of effective points narrows rapidly, and the attenuation rate of remote sensing performance surges drastically (exceeding 50%). Effective points within the 300 m range become discretized, leading to a severe degradation in short-range, high-precision underwater remote sensing capabilities.

This threshold (150 m), derived not only from the critical observation point in simulations (~185 m) but also integrated with safety redundancy for dynamic environments, provides a scientific and reliable quantitative basis for designing depth combinations of underwater remote sensing platforms with heterogeneous transceiver depths.

## 4. Discussion

### 4.1. Validity and Physical Mechanism of Reciprocity Under Seamount Topography

Quantitative analysis (see Figure 11) reveals that, while local transmission loss deviations were observed at the edges of shadow zones and regions of intense multipath interference, the maximum absolute error remained within 1.76 dB, with a relative error below 1.2%.

Physically, these minor deviations do not stem from non-reciprocal properties of the medium but are primarily attributed to path sampling differences arising from numerical discretization. Near steep terrain, a finite bundle of rays cannot perfectly cover all high-order reflection and diffraction components in both bidirectional paths. This further validates the engineering applicability of the acoustic reciprocity theorem in complex marine environments, providing theoretical support for simplifying signal processing in underwater acoustic detection networks.

### 4.2. Mechanisms Influencing Buoy Remote Sensing Performance

In this study, transceiver depth affects performance through ray propagation paths and energy attenuation laws. The underlying mechanisms are summarized as follows:

1. Gradient Effect of Sound Speed Profile:
  (1) Shallow Layer: The positive gradient refracts rays toward the surface, causing rapid energy decay and limiting the remote sensing range.
  (2) Mid-Layer: The negative gradient bends rays inward, stabilizing propagation paths and minimizing attenuation. This forms the optimal detection interval, creating favorable conditions for efficient remote sensing.
  (3) Deep Layer: Influenced by topography, reflection losses increase with depth, causing slight range attenuation.
2. Superposition Effect of Transceiver Depths:
  (1) Co-Depth: The Source-to-Target and Receiver-to-Target ray paths highly overlap, maximizing the superposition of low-loss zones and ensuring broad, continuous effective coverage.
  (2) Bistatic: Depth separation causes path divergence, narrowing the superposition range, reducing effective points, and degrading performance.
3. Coupling Effect of Topography:

Undulating features such as seamounts and trenches alter ray directions, generating shadow zones in which transmission loss spikes abruptly. This leads to discontinuous effective point distributions, which is a primary cause of the discretization observed in the 0~300 m layer under bistatic configurations, impacting the spatial continuity of remote sensing data.

### 4.3. Engineering Applicability of Deployment Schemes

The proposed deployment schemes are directly applicable to underwater acoustic detection scenarios involving 300 Hz low-frequency buoys, horizontally inhomogeneous sound speeds, and undulating seabed topography. The proposed deployment schemes are only directly applicable to deep-sea complex environments with water depth >5000 m, involving horizontally inhomogeneous sound speeds and undulating seabed topography. This method is based on the high-frequency condition of ray theory satisfied by 300 Hz in deep-sea environments and is not applicable to shallow-sea scenarios. For short-range, high-precision tasks (e.g., underwater security, precise target localization): transceiver depth separation must be strictly controlled (≤150 m) to ensure data integrity and accuracy. For large-scale monitoring and long-range search: priority should be given to co-depth deployment in the 160~350 m range to maximize detection coverage.

In practical engineering applications, adaptations must be made based on buoy frequency and environmental conditions:

1. High-Frequency Buoys (>1 kHz): Due to faster attenuation and shorter ranges, shallow deployment is recommended based on simulation results. This balances ease of deployment with the need for short-range, high-resolution remote sensing.
2. Medium-Frequency Buoys (500 Hz~1 kHz): With slower attenuation and strong long-range capabilities, deep deployment is advisable to further extend detection ranges, suitable for medium-to-long-range tasks.

The simulation data in this paper is based on the internationally authoritative HYCOM hydrological data and ETOPO1 topographic data, and the Bellhop model is a standard ray model for underwater acoustic simulation. The acoustic reciprocity verification shows that the maximum relative error of TL is less than 1.2%, which proves the reliability of the simulation results. Due to experimental conditions, sea trial verification has not been carried out temporarily, and it will be carried out in the follow-up research.

## 5. Conclusions

Based on simulations at 300 Hz using the Bellhop model, this study verified the applicability of the acoustic reciprocity theorem in deep-sea (>5000 m) complex environments in a specific sea area (122.467°~123.595° E, 22.33° N), providing a theoretical basis for data processing and model simplification in deep-sea underwater acoustic detection. We systematically analyzed the impact of co-depth and bistatic depth configurations on buoy performance and proposed optimized deployment strategies. The main conclusions are as follows:

1. Validity of Reciprocity: The acoustic reciprocity theorem holds fundamentally in complex environments with horizontal sound speed inhomogeneity and undulating topography. The maximum difference between Target-to-Receiver and Receiver-to-Target transmission losses is only 1.76 dB, which is negligible relative to the total loss. This enables simplified calculation of transmission losses, significantly enhancing data processing efficiency.
2. Optimal Co-Depth Interval: Under co-depth deployment, performance varies non-linearly with depth. The 160~350 m interval is optimal, offering stable ray paths, minimal boundary losses, maximum detection ranges over 100 km, and more than 500,000 effective points. This is the preferred depth for long-range remote sensing.
3. Critical Threshold in Bistatic Configurations: Performance is strongly negatively correlated with transceiver depth separation. A critical threshold of 150 m was identified. Exceeding this value causes effective points to transition from continuous to discrete patches. For mid-layer sources (161.98~231.40 m), the detection range drops by over 50%, severely degrading short-range coverage.
4. Layer-Dependent Suitability: (Note: corrected based on context) Shallow sources are suitable for short-range tasks with loose depth constraints; mid-layer sources require precise co-depth deployment for high-quality data; deep sources are robust for medium-to-long-range bistatic operations.

Based on these findings, we propose the following optimization strategies:

1. Priority Strategy: For long-range, large-scale tasks, adopt co-depth deployment in the 160~350 m range to leverage optimal performance.
2. Alternative Strategy: If co-depth deployment is unfeasible, strictly limit transceiver depth separation to <150 m, ensuring both source and receiver remain within the 160~350 m optimal window to maintain performance.
3. Special Strategy: For short-range, low-precision tasks, shallow deployment is permissible with larger depth separations (<500 m), balancing operational convenience with mission requirements.

Deployment Considerations:

(1) Pre-deployment monitoring of local sound speed profiles and bathymetry is essential to define the specific optimal depth window.
(2) During deployment, the depths of all nodes should be kept consistent, or the depth difference must be maintained at ≤150 m. This threshold incorporates a safety margin to account for dynamic environmental conditions, thereby preventing blind spots in the remote sensing coverage between nodes.
(3) For mid-layer sources, strict control of positioning accuracy is required to prevent depth deviations from exceeding the threshold.

This study provides a scientific basis and engineering reference for buoy depth deployment in marine engineering, offering significant practical value for improving remote sensing accuracy, optimizing observation system strategies, and constructing efficient, reliable three-dimensional marine observation networks.

## Figures and Tables

**Table 1:** Number of spatial points and detection distance for different transceiver depth combinations.

Source Depth (m)	Receiver Depth (m)	Depth Separation (m)	Total Points	Tactical Points (0~300 m)	Number of Sound Source Depth Points	Max Range (km)	Sum of Transmission Loss (dB)
23.14	23.14	0	498,362	14,659	393	58.31	159.86
	69.42	46.28	534,620	14,431	270	49.60	158.62
	161.98	138.84	522,905	14,285	251	48.76	159.32
	231.40	208.26	516,862	13,443	233	48.04	158.11
	347.10	323.96	514,473	13,942	256	48.04	155.75
	578.50	555.36	501,020	13,913	253	47.95	158.67
69.42	69.42	0	555,955	16,801	415	49.74	151.72
	161.98	92.56	536,570	15,041	368	49.37	154.37
	231.40	161.98	536,505	14,211	351	49.37	154.24
	347.10	277.68	535,663	14,491	325	49.37	155.54
	578.50	509.08	520,246	15,025	365	49.37	158.46
161.98	161.98	0	539,375	15,771	362	114.54	153.78
	231.40	69.52	528,310	14,739	310	114.14	157.80
	347.10	185.12	521,184	14,681	309	114.14	157.07
	578.50	416.52	500,282	14,598	294	57.18	157.36
231.40	231.40	0	561,586	16,959	513	110.75	159.84
	347.10	115.70	530,057	15,612	386	108.35	159.84
	578.50	347.40	508,686	14,653	328	108.05	153.71
347.10	347.10	0	577,637	16,199	624	106.33	159.29
	578.50	231.40	528,872	14,831	450	99.20	159.22
578.50	578.50	0	616,947	15,712	670	99.59	159.06

## Data Availability

The topographic data utilized in this study are publicly available from the ETOPO1 Global Relief Model database (https://www.ngdc.noaa.gov/mgg/global/) (accessed on 15 August 2025). Hydrological data were acquired from the HYCOM (Hybrid Coordinate Ocean Model) reanalysis dataset, which can be accessed via https://hycom.org/data/ (accessed on 11 March 2025). The simulation datasets generated during the current study (including transmission loss simulation results and maximum sonar detection range data) are not publicly available due to internal research data management policies but are available from the corresponding author (Pingbo Wang) upon reasonable request.
